# Supplementary material for: A direct interareal feedback-to-feedforward circuit in primate visual cortex
Source: Nat Commun. 2021 Aug 13;12:4911. doi: 10.1038/s41467-021-24928-6 (PMC8363744; doi:10.1038/s41467-021-24928-6)
Supplement: Supplementary file 1 — Supplementary Information [file 41467_2021_24928_MOESM1_ESM.pdf]

Supplementary Table 1

Injection parameters and survival times across cases

	TRIO CASES			CONTROL CASES	
**Case No.**	**MK405**	**MK382**	**MK379**	**MK380**	**MK381**
**Sex**	F	F	F	F	F
**Weight** (kg)	4.1	3	2.9	3	3.5
**Number of Injections**					
AAV	3	3	2	1	1
RV	3	3	2	1	1
CAV	2	2	1	0	0
**Survival Times** (days)					
Post-AAV injections	21	24	21	21	33
Post-CAV injections	8	11	2	–	–
Post-RV injections	10	9	12	9	7
**Tot. Volume per injection** (nl)					
AAV	510	225	510	480	225
CAV	480	270	500	–	–
RV	600	300	600	300	300
**AAV-TVA:AAV-oG Ratio**					
	3:7	3:7	1:1	3:7	3:7
**Depth of each injection** (μm)					
V1	600 +1200	400 + 800	400 + 800	–	–
V2	700 +1000	700	700	–	–
Motor Cortex	–	–	–	600+1000	400 + 800

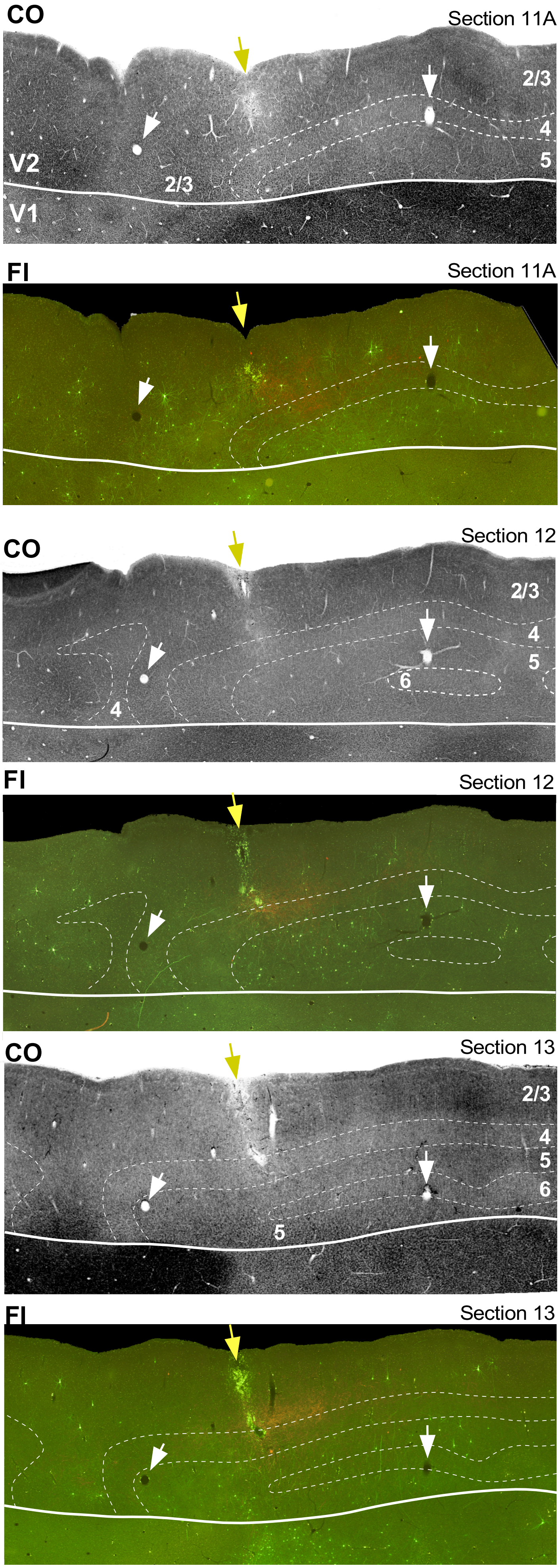

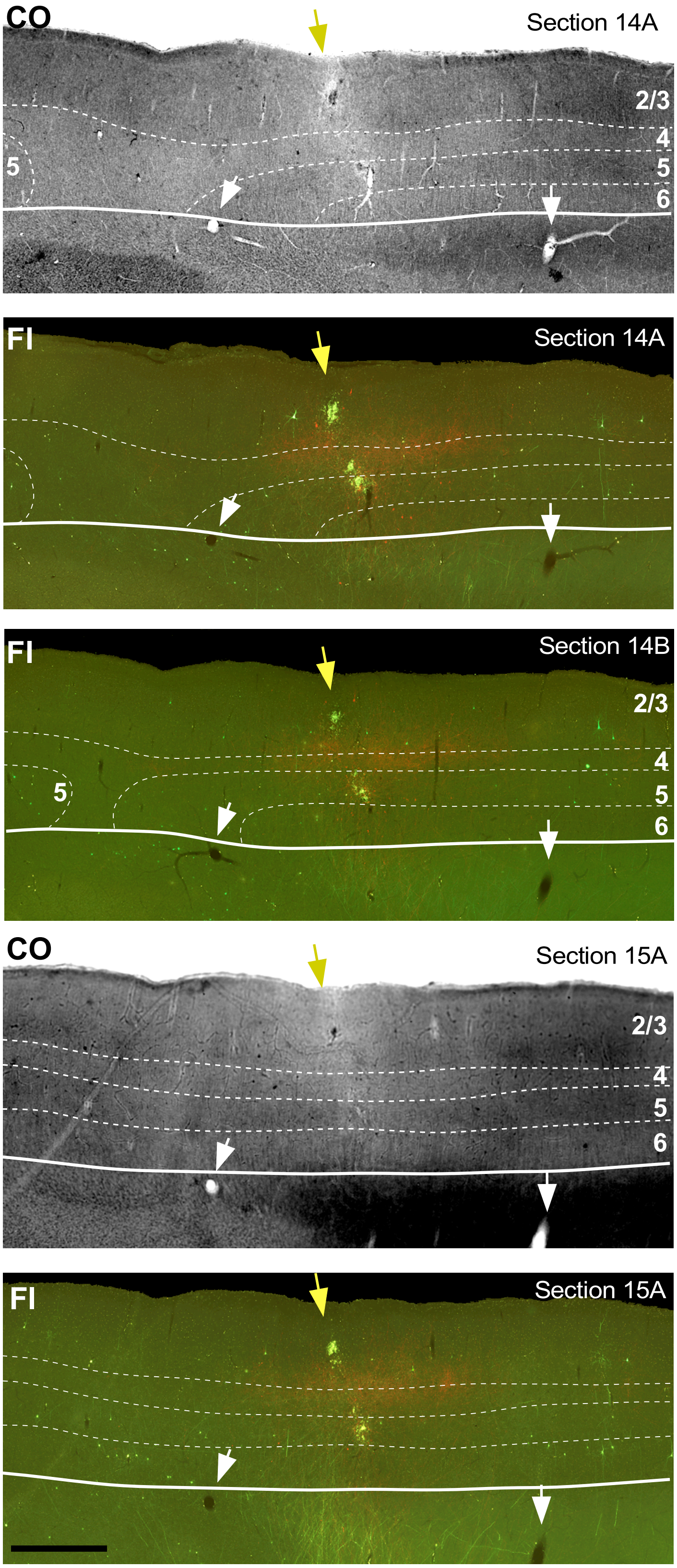

Supplementary Figure 1

Injection site and GFP-labeled neurons in V2. Case MK405. Sequence of tangential sections (11A most superficial) through V2 and part of V1. For all, but Sect 14B, the same section is shown stained for CO and imaged for GFP and mCherry fluorescence (*Fl*). *Dashed contours* delineate cortical layers (as indicated on CO sections); *solid contour:* V1/V2 border. V2 L4 is pale in CO and L5 is darker than L6. V1 afferents (*red fibers*) terminate in L3B-4 nearby the injection site (*yellow arrow*). *White arrows* point at same blood vessels across sections. Scale bar applies to all panels. Results are representative of 3 independent TRIO experiments made in 3 different animals. Scale bar: 1mm valid for all panels.

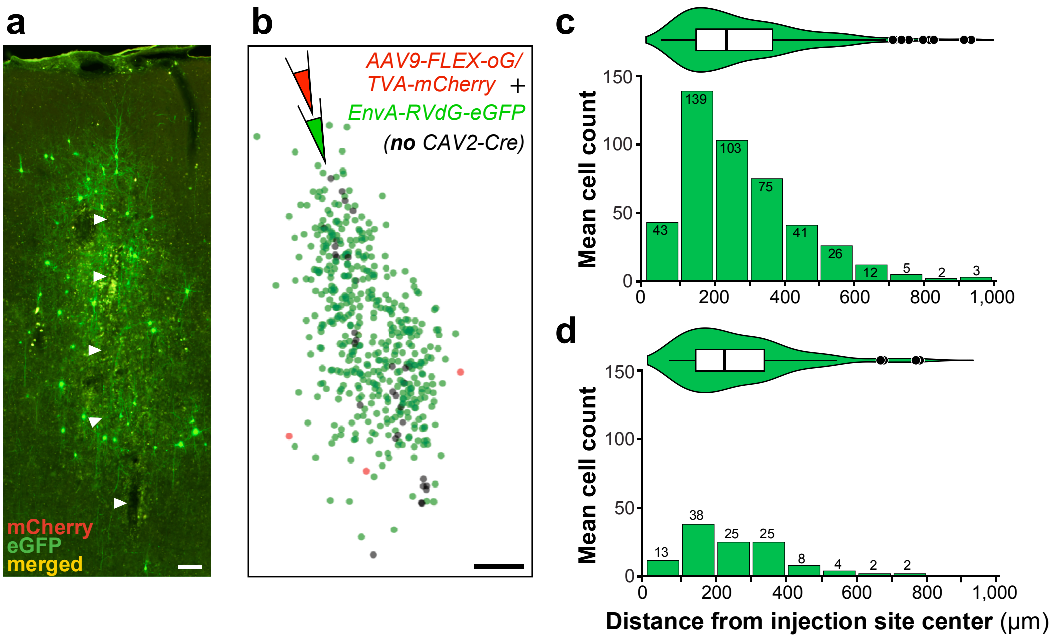

Supplementary Figure 2

Control experiments for TRIO.

(a) Case MK381. Fluorescent image of a single sagittal tissue section through the motor cortex, showing local Cre-independent expression of GFP in a control experiment in which CAV2-Cre was omitted from the TRIO injection protocol. The protocol was otherwise as described in Fig.1a. The section was imaged for mCherry and GFP, and the two channels were merged. Only GFP-labeled cells are visible in this section. *Arrowheads* point to the pipette track, which was defined as the center of the viral injection site. Results in (a) are representative of 2 independent controls made in 2 different animals. Scale bar: 100μ m. **(b)** Plots of all GFP- (*green dots*) labeled cells across all sections containing GFP label (n=19, 60μm-thick sections), for the same control case. *Red dots* are plots of mCherry-labeled cells; these were virtually absent, amounting to a total of 3 cells in this case, and 1 cell in the second control case. *Black dots* mark the pipette track (center of injection site). Scale bar: 500 μm. **(c)** Distribution of GFP-labeled cell distances from the center of the injection site, for the same control case (MK381), plotted on the same axis as a histogram (*Bottom*) and as a violin plot (*Top*; Minima=4.4μm, Maxima=935.6μm, Median (*black line*)=231.4μm, 1QR (lower bound of box)=145.7μm, 3QR (upper bound of box)=364.9μm, Mean=273.6μm). We counted a total of 449 GFP-labeled cells of which only 89 (~20%) were located at distances >400 μm from the injection center. **(d)** Same as in (c) but for the second control case (MK380) which received larger AAV injection volumes than case MK381 (see Supplementary Table 1). Despite the larger injection volumes, this case resulted in smaller number of GFP-labeled cells (117), of which only 16 (~14%) were located at distances >400 μm from the injection center. We found no GFP-labeled cells outside the injected cortical area, indicating that all inter-areal inputs are specific, i.e. dependent on CAV2-Cre. Violin plot: Minima=63.5μm, Maxima=778.6μm, Median=220.9μm, 1QR=139.4μm, 3QR=334.8μm, Mean=255μm.

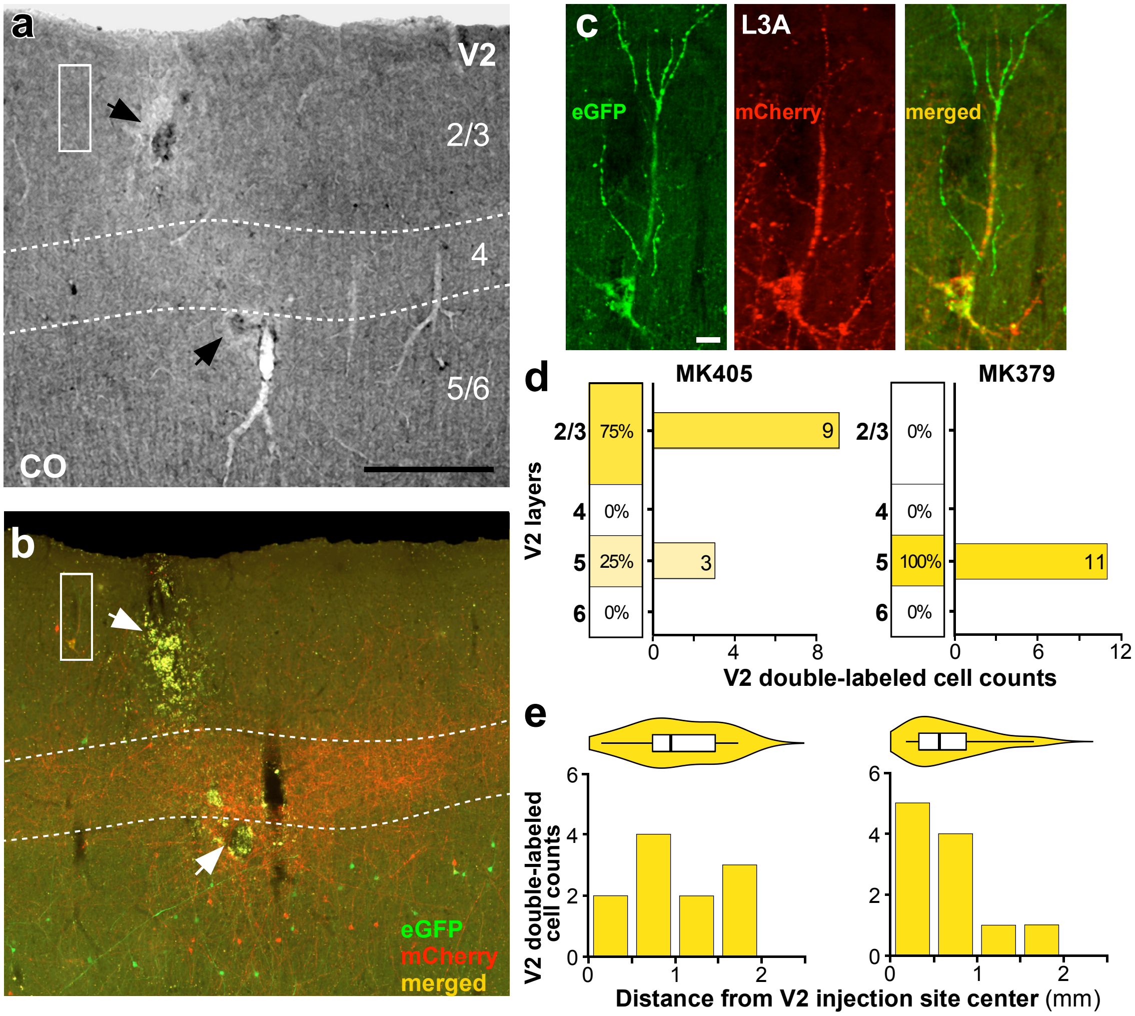

Supplementary Figure 3

Double-labeled cells in V2.

(a) Case MK405. Image of a V2 tissue sections stained for CO, revealing all layers. *Arrows* here and in (b) point to the V2 injection site, and *dashed contours* delineate the boundaries of L4. Scale bar: 500 µm (valid also for b). **(b)** Image of a V2 section immediately adjacent to the one in (a), imaged for mCherry and GFP and merged. Notice red fiber label in L4 and lower L3 representing m-Cherry labeled V1->V2 axon terminals. The region inside the *white box* is shown at higher magnification in (c). **(c)** A double-labeled pyramidal cell in V2 L3A shown under GFP (*Left*) or mCherry (*Middle*) fluorescence, and merged (*Right*). Scale bar: 20 µm. **(d)** Percent and number of V2 double-labeled cells across layers for each of the two cases that had double-labeled cells in V2. **(e)** Distribution of double-labeled cell distances from the center of the V2 injection site plotted on the same axis as a histogram (*Bottom*) and as a violin plot (*Top*), for each case. LEFT violin plot: Minima=146.3µm, Maxima=1739µm, Median (*black line*)=952.3µm, 1QR (lower bound of box)=738.4µm, 3QR (upper bound of box)=1468.5µm, Mean=1019.3µm, n=11 cells. RIGHT violin plot: Minima=179.5µm, Maxima=1653.1µm, 1QR=328.3µm, 3QR=874.5µm, Mean=670.6µm, n=11 cells.

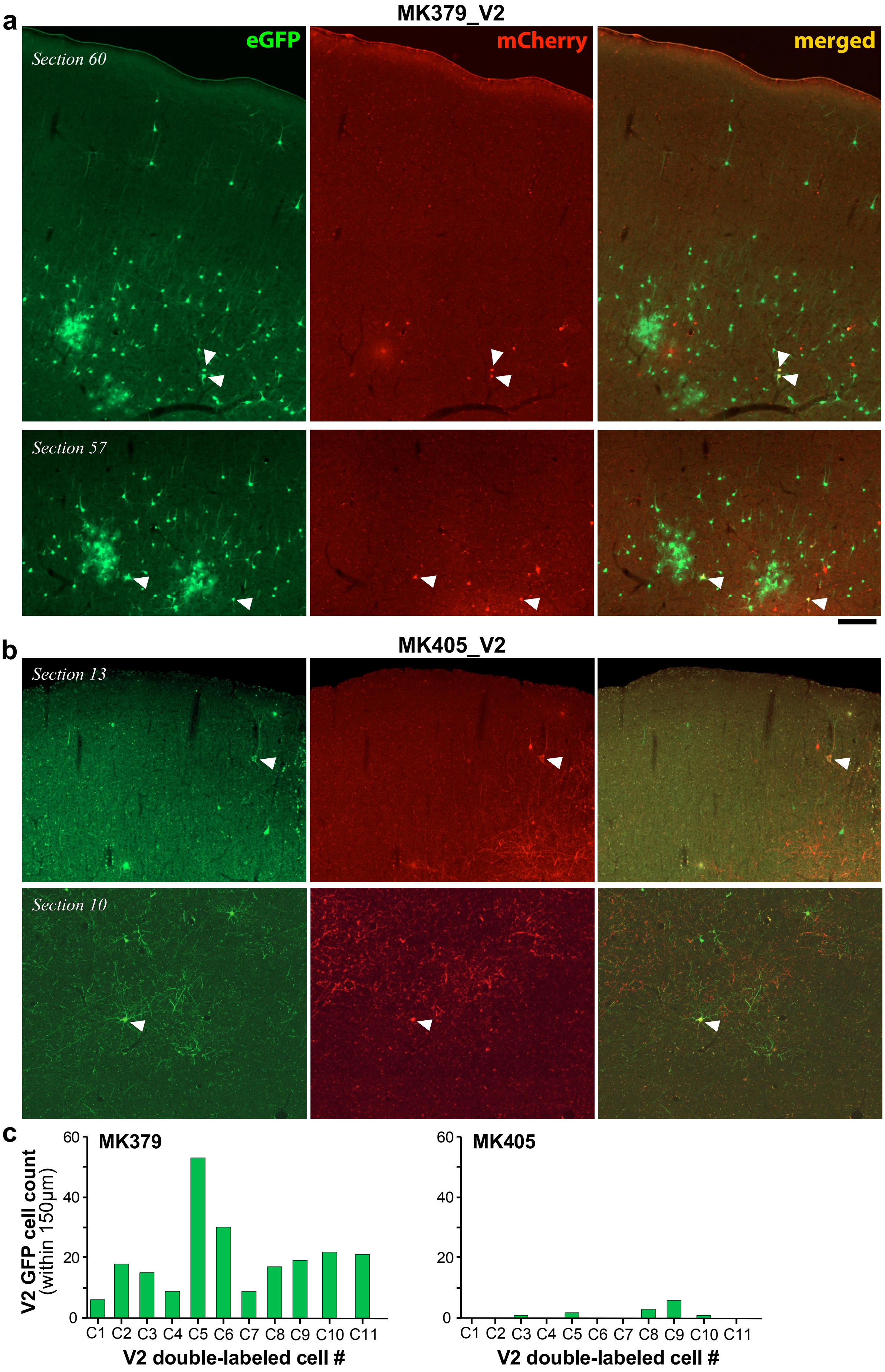

Supplementary Figure 4

Pattern of local GFP label surrounding V2 double-labeled cells.
(a) Case MK379. Two representative sections through V2 imaged under GFP (*Left*) and mCherry (*Middle*) fluorescence, and merged (*Right*), showing double-labeled cells in V2 (*arrowheads).* Scale bar: 200 µm valid for (a-b). **(b)** Same as in (a), but for case MK405. In (a) many GFP-labeled neurons surround the double-labeled cells. In contrast, in (b), double-labeled cells are isolated and the nearest GFP-labeled cells are located several hundred microns away. **(c)** Number of GFP-labeled V2 cells within 150 µm radius of each double-labeled cell (C1 to C11) in V2 for each case.
