# Supplementary material for: A direct interareal feedback-to-feedforward circuit in primate visual cortex
Source: Nat Commun. 2021 Aug 13;12:4911. doi: 10.1038/s41467-021-24928-6 (PMC8363744; doi:10.1038/s41467-021-24928-6)
Supplement: Supplementary file 3 — Reporting Summary [file 41467_2021_24928_MOESM3_ESM.pdf]

nature research

Corresponding author(s): Alessandra Angelucci

Last updated by author(s): May 21, 2021

Reporting Summary

Nature Research wishes to improve the reproducibility of the work that we publish. This form provides structure for consistency and transparency in reporting. For further information on Nature Research policies, see our Editorial Policies and the Editorial Policy Checklist.

Statistics

For all statistical analyses, confirm that the following items are present in the figure legend, table legend, main text, or Methods section.

n/a	Confirmed	
☐	☒	The exact sample size (*n*) for each experimental group/condition, given as a discrete number and unit of measurement
☒	☐	A statement on whether measurements were taken from distinct samples or whether the same sample was measured repeatedly
☒	☐	The statistical test(s) used AND whether they are one- or two-sided<br>*Only common tests should be described solely by name; describe more complex techniques in the Methods section.*
☒	☐	A description of all covariates tested
☒	☐	A description of any assumptions or corrections, such as tests of normality and adjustment for multiple comparisons
☐	☒	A full description of the statistical parameters including central tendency (e.g. means) or other basic estimates (e.g. regression coefficient) AND variation (e.g. standard deviation) or associated estimates of uncertainty (e.g. confidence intervals)
☒	☐	For null hypothesis testing, the test statistic (e.g. *F*, *t*, *r*) with confidence intervals, effect sizes, degrees of freedom and *P* value noted<br>*Give P values as exact values whenever suitable.*
☒	☐	For Bayesian analysis, information on the choice of priors and Markov chain Monte Carlo settings
☒	☐	For hierarchical and complex designs, identification of the appropriate level for tests and full reporting of outcomes
☒	☐	Estimates of effect sizes (e.g. Cohen's *d*, Pearson's *r*), indicating how they were calculated

Our web collection on statistics for biologists contains articles on many of the points above.

Software and code

Policy information about availability of computer code

Data collection	VDAQ Version 2.5, Optical Imaging LTD<br>Zen (blue edition) Version 2.6 Blue Software, Carl Zeiss Microscopy GmbH, for image acquisition and analysis, and for stitching image tiles (Stiching Algorithm)
Data analysis	Adobe Photoshop Versions 6, 2019, and 2020, Adobe Inc.<br>Neurolucida Software Version 11.09 64bit (Microbrightfield Bioscience, VT, USA)<br>RStudio desktop Version 1.4.1103

For manuscripts utilizing custom algorithms or software that are central to the research but not yet described in published literature, software must be made available to editors and reviewers. We strongly encourage code deposition in a community repository (e.g. GitHub). See the Nature Research guidelines for submitting code & software for further information.

Data

Policy information about availability of data

All manuscripts must include a data availability statement. This statement should provide the following information, where applicable:
- Accession codes, unique identifiers, or web links for publicly available datasets
- A list of figures that have associated raw data
- A description of any restrictions on data availability

The data that support the findings of this study are available from the corresponding author upon reasonable request. Source data are provided with this paper.

Field-specific reporting

Please select the one below that is the best fit for your research. If you are not sure, read the appropriate sections before making your selection.

☒ Life sciences ☐ Behavioural & social sciences ☐ Ecological, evolutionary & environmental sciences

For a reference copy of the document with all sections, see nature.com/documents/nr-reporting-summary-flat.pdf

Life sciences study design

All studies must disclose on these points even when the disclosure is negative.

Sample size	In primate neuroanatomical studies, power analyses to estimate sample sizes are not standard and, in most instances, not possible, as they require knowledge of effect size, which is typically unknown. Reproducibility is demonstrated by reproducing results in at least 3 animals. Most neuroanatomical studies of the kind we have performed in this study report statistically rigorous results with 3 tracer injections per area and/or compartment. This is what we have done in this study. We have collected data from viral injections made in 3 animals and counted resulting labeled cells. As the results across the 3 animals were consistent, we determined that this sample size was sufficient. Sample size was, thus determined on the basis of significantly similar results across the 3 cases.
Data exclusions	We excluded from analysis 3 cases which did not result in labeled cells due to lack of overlap between the V1 and V2 injections, and 1 case in which the V2 injection spilled into V1. These exclusion criteria were pre-established at the start of the study.
Replication	We replicated each experiment independently 3 times. Details about each experiment and their replication are reported in each relevant figure legend.
Randomization	Not relevant because all 3 animals received the same "treatment".
Blinding	Not relevant because all amimals received the same "treatment" (injections in the same areas).

Reporting for specific materials, systems and methods

We require information from authors about some types of materials, experimental systems and methods used in many studies. Here, indicate whether each material, system or method listed is relevant to your study. If you are not sure if a list item applies to your research, read the appropriate section before selecting a response.

Materials & experimental systems

n/a	Involved in the study	
☐	☒	Antibodies
☒	☐	Eukaryotic cell lines
☒	☐	Palaeontology and archaeology
☐	☒	Animals and other organisms
☒	☐	Human research participants
☒	☐	Clinical data
☒	☐	Dual use research of concern

Methods

n/a	Involved in the study	
☒	☐	ChIP-seq
☒	☐	Flow cytometry
☒	☐	MRI-based neuroimaging

Antibodies

Antibodies used	- Mouse Anti-Calbindin D-28k, monoclonal, Swant, Switzerland, Catalogue No. 300, Lot No. 07 (F)<br>- Alexa Fluor® 647-conjugated AffiniPure Donkey Anti-Mouse IgG (H+L), Jackson ImmunoResearch, PA,USA,, Catalogue No: 715-605-150, Lot No.: 139394
Validation	Anti-Calbindin D-28K (from manufacturer website): The antibody was evaluated for specificity and potency: a) by indirect immunofluorescent or immunoperoxidase labelling as well as Biotin-Avidin labeling of cryostat or vibratome-sections of 4 % paraformaldehyde-fixed tissue; b) by immunoenzymatic labelling of immunoblots; c) by radioimmunoassay.<br>Immunohistochemistry on Calbindin D-28k knock-out mice: Antibody CB300 immunolabels a subpopulation of neurons in the normal brain with high efficiency but does not stain in the brain of calbindin D-28k knock out mice. Suggested Working dilution for immunohistochemistry: 1:5000-1:10,000

Animals and other organisms

Policy information about studies involving animals; ARRIVE guidelines recommended for reporting animal research

Laboratory animals	Macaca Fasicularis, 3 females between the ages of 2 and 5 years old.

Wild animals	The study did not involve wild animals.
Field-collected samples	The study did not involve samples collected from the field.
Ethics oversight	University of Utah Institutional Animal Care and Use Committee

Note that full information on the approval of the study protocol must also be provided in the manuscript.
